# FloodCastBench: A Large-Scale Dataset and Foundation Models for Flood Modeling and Forecasting

**DOI:** 10.1038/s41597-025-04725-2

**Published:** 2025-03-12

**Authors:** Qingsong Xu, Yilei Shi, Jie Zhao, Xiao Xiang Zhu

**Affiliations:** 1 Data Science in Earth Observation, Technical University of Munich, Munich, 80333 Germany; 2 Munich Center for Machine Learning, Munich, 80333 Germany; 3 School of Engineering and Design, Technical University of Munich, Munich, 80333 Germany

**Keywords:** Hydrology, Natural hazards

## Abstract

Effective flood forecasting is crucial for informed decision-making and emergency response. Existing flood datasets mainly describe flood events but lack dynamic process data suitable for machine learning (ML). This work introduces the FloodCastBench dataset, designed for ML-based flood modeling and forecasting, featuring four major flood events: Pakistan 2022, UK 2015, Australia 2022, and Mozambique 2019. FloodCastBench details the process of flood dynamics data acquisition, starting with input data preparation (e.g., topography, land use, rainfall) and flood measurement data collection (e.g., SAR-based maps, surveyed outlines) for hydrodynamic modeling. We deploy a widely recognized finite difference numerical solution to construct high-resolution spatiotemporal dynamic processes with 30-m spatial and 300-second temporal resolutions. Flood measurement data are used to calibrate the hydrodynamic model parameters and validate the flood inundation maps. FloodCastBench provides comprehensive low-fidelity and high-fidelity flood forecasting datasets specifically for ML. Furthermore, we establish a benchmark of foundational models for neural flood forecasting using FloodCastBench, validating its effectiveness in supporting ML models for spatiotemporal, cross-regional, and downscaled flood forecasting.

## Background & Summary

Flooding is a recurrent and pervasive natural hazard, consistently causing substantial damage to both human lives and infrastructure on a global scale^1,2^. This highlights the urgent need for reliable flood forecasting systems^1,2^. Accurate spatiotemporal flood forecasts enable the timely dissemination of crucial disaster information to government decision-makers, emergency responders, and vulnerable communities, thereby enhancing preparedness and significantly mitigating flood-related risks^3,4^.

With the rapid advancements in machine learning (ML)^5–7^, the fusion of ML attributes like rapid inference, and resolution flexibility, with the interpretability and transferability of physical models, presents the potential for precise, real-time flood forecasting^4,8^. However, existing datasets, such as the geocoded disaster dataset^9^, the database of flood fatalities from the Euro-Mediterranean region^10^, the gridded dataset of Earth’s floodplains at 250-m resolution^11^, the database of European coastal flood maps^12^, and the global database of flood events based on social media^13^, predominantly focus on the multi-modal descriptions of flood events. These datasets encompass aspects such as flood locations^9^, flood fatalities^10^, flood inundation extents^11,12^, and flood descriptions based on social media^13^. Presently, there is an evident dearth of datasets that capture the dynamic processes of flooding suitable for ML. These datasets are characterized by high spatiotemporal resolution, multi-scale and multi-scene attributes, as well as the inclusion of multi-source inputs^4,14^.

Bear these concerns in mind, this work introduces the FloodCastBench dataset, a large-scale dataset developed to capture the dynamics of floods, with an emphasis on the spatiotemporal evolution of water depth and the extent of inundated areas. Specifically, we first analyze the global map of flood occurrences spanning from 1985 to 2023, selecting four large flood events as study areas: Pakistan flood 2022, UK flood 2015, Australia flood 2022, and Mozambique flood 2019. The FloodCastBench dataset acquisition process consists of three stages: data preparation, data production, and data calibration. During the data preparation phase, we gather relevant input data to initialize the hydrodynamic model and collect flood measurement data to support hydrodynamic modeling and parameter calibration. In the data production stage, a widely recognized numerical solution (finite difference) is deployed to construct high-resolution spatiotemporal dynamic processes with a spatial resolution of 30 m and a temporal resolution of 300 seconds. Flood measurement data are used to calibrate the hydrodynamic model parameters and validate the final flood inundation maps. Finally, the FloodCastBench dataset encompasses three components: flood dynamic results with a spatial resolution of 30 m  × 30 m and a temporal resolution of 300 seconds, spatially resampled flood dynamic results for low-fidelity and high-fidelity flood forecasting based on ML models, and flood dynamic results for cross-regional transferability of ML models. Furthermore, based on the constructed FloodCastBench dataset, we have established a benchmark of foundational models for neural flood forecasting. This benchmark provides a solid foundation and detailed evaluation criteria for foundational models in low-fidelity and high-fidelity flood forecasting and cross-regional flood forecasting tasks. Additionally, it validates the effectiveness of the FloodCastBench dataset in supporting ML models for spatiotemporal flood forecasting, cross-regional, and downscaled flood forecasting.

Our contributions can be summarized as follows:

- FloodCastBench provides detailed information about the process of flood dynamics data acquisition, including data preparation, data production, and data calibration.
- FloodCastBench constitutes the pioneering flood forecasting dataset specifically for ML.
- FloodCastBench includes benchmark experiments of foundational models for large-scale, cross-regional, and downscaled flood forecasting based on ML.

## Methods

### Study Regions

**Fig. 1:**
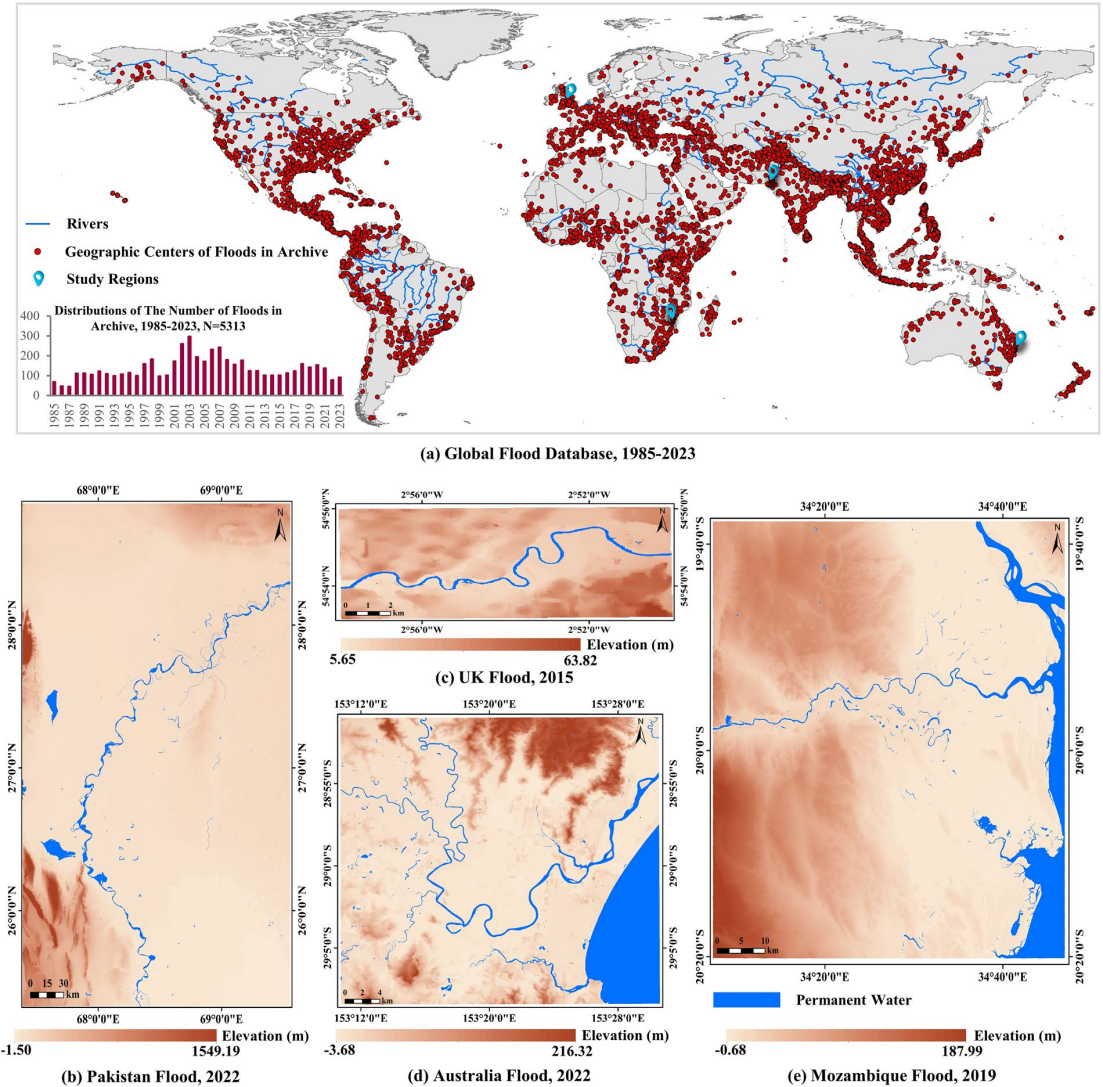
Global flood database and locations of the study areas.

To gain a comprehensive understanding of global flood distribution, we utilize Dartmouth Flood Observatory Dataset^15^ and Global Disaster Alert and Coordination System^16^ to compile the global map of flood occurrences spanning from 1985 to 2023, documenting a total of 5,313 significant flood events, as depicted in Fig. 1(a). This figure showcases the persistent high occurrence of major flood events annually, widely dispersed across the globe. Thus, effective flood forecast assumes heightened significance. We select four large-scale flood events to create a real-world flood forecasting dataset. The visual representations of Digital Elevation Models (DEMs) and river networks for these events are shown in Fig. 1(b),(c),(d), and (e), respectively. These events are as follows:

- Pakistan Flood 2022. During the 2022 summer monsoon season, Pakistan experienced catastrophic flooding triggered by intense and prolonged rainfall, making it one of the most severe disasters in the country’s history. This flood event affected nearly one-third of Pakistan’s population, displacing 32 million people and causing 1,486 fatalities, including 530 children, with economic losses exceeding $30 billion^17^. The study area focuses on the regions most profoundly impacted by this flooding, covering the southern provinces of Balochistan, Sindh, and Punjab, with a total land area of 85,616.5 square kilometers^4^. The Indus River Basin, a crucial drainage system, plays a fundamental role in shaping the hydrological characteristics of the region. A notable change occurred between August 18 and August 31, 2022, marked by significant increases in flood coverage within the Pakistan study area^4^. Consequently, we implement the flood simulation for this 14-day period, totaling 1,209,600 seconds.
- UK Flood 2015. The UK Flood region, situated in northwest England, is dominated by the 145-kilometer-long River Eden, flowing from southeast to northwest. This catchment area comprises four principal tributaries: Caldew, Petteril, Eamont, and Irthing. Due to its steep topography upstream, the Eden catchment experiences frequent fluvial flooding, necessitating rapid response mechanisms. The downstream area, particularly Carlisle, has a history of significant flood occurrences, notably the devastating event of December 2015, which inflicted substantial damage^18^. The study region covers approximately 135.5 square kilometers. The event was caused by the intense rainfall from 4 to 7 December 2015 (3 days).
- Australia Flood 2022. The flooding in eastern Australia, which commenced in February 2022, exacerbated following additional heavy rainfall. Authorities documented widespread flooding in Queensland and New South Wales, resulting in the evacuation of thousands of residents and damage to numerous homes. The Richmond river basin, serving as a vital drainage system, significantly influences the hydrology of the study area^19^. Encompassing regions in Ballina, the study area spans a total land area of 1,361.3 square kilometers. The flood simulation period spans from February 20 to March 02, 2022, leveraging rainfall records and SAR-based flood observations.
- Mozambique Flood 2019. Tropical Cyclone Idai struck the coastal city of Beira, Sofala Province, Central Mozambique on 14 March 2019, unleashing heavy rainfall and powerful winds persisting for over a week. Consequently, the Pungwe and Buzi Rivers, along with lakes, overflowed, inundating low-lying areas^20^. This flood caused 4,000 houses to be damaged or inhabitable, about 1,600 people were injured, and 603 people were killed in Mozambique, Zimbabwe, and Malawi^21^. Encompassing the regions within Beira, Mozambique, the study area spans an extensive land area of 6,190.9 square kilometers. The flood simulation period extends from March 14 to March 20, 2019, according to rainfall records and SAR-based flood observations.

### Data acquisition

The data acquisition and production process comprises three key stages: data preparation, data production, and data calibration. In the data preparation phase, two main aspects of data are prepared. First, relevant input data for flood simulation are gathered to initialize the hydrodynamic model. These data encompass topographical information (DEM), land use and land cover data, and rainfall data. Second, flood measurement data are collected to facilitate hydrodynamic modeling and input parameter calibration. These data include SAR-based flood maps and surveyed flood outlines. In the data production stage, traditional finite difference solvers are predominantly employed to resolve the dynamics of the flood process. Finally, in the data calibration stage, input parameters (terrain and Manning coefficient), initial conditions, and boundary conditions of the hydrodynamic model are fine-tuned to achieve excellent alignment between the resultant hydrodynamic outcomes and the flood measurement data.

#### Data preparation

##### Topography data

A high-resolution (30 m) forest and buildings removed Copernicus digital elevation model (FABDEM)^22^ from COPDEM30 is required for flood simulation. The data are provided in GeoTIFF format, with each file segmented into 1  × 1 degree tiles. These files are organized into zipped folders, each covering a 10  × 10 degree area. To obtain the relevant terrain data, we crop the files to match the boundaries of the study regions.

##### Land use and land cover data

**Fig. 2:**
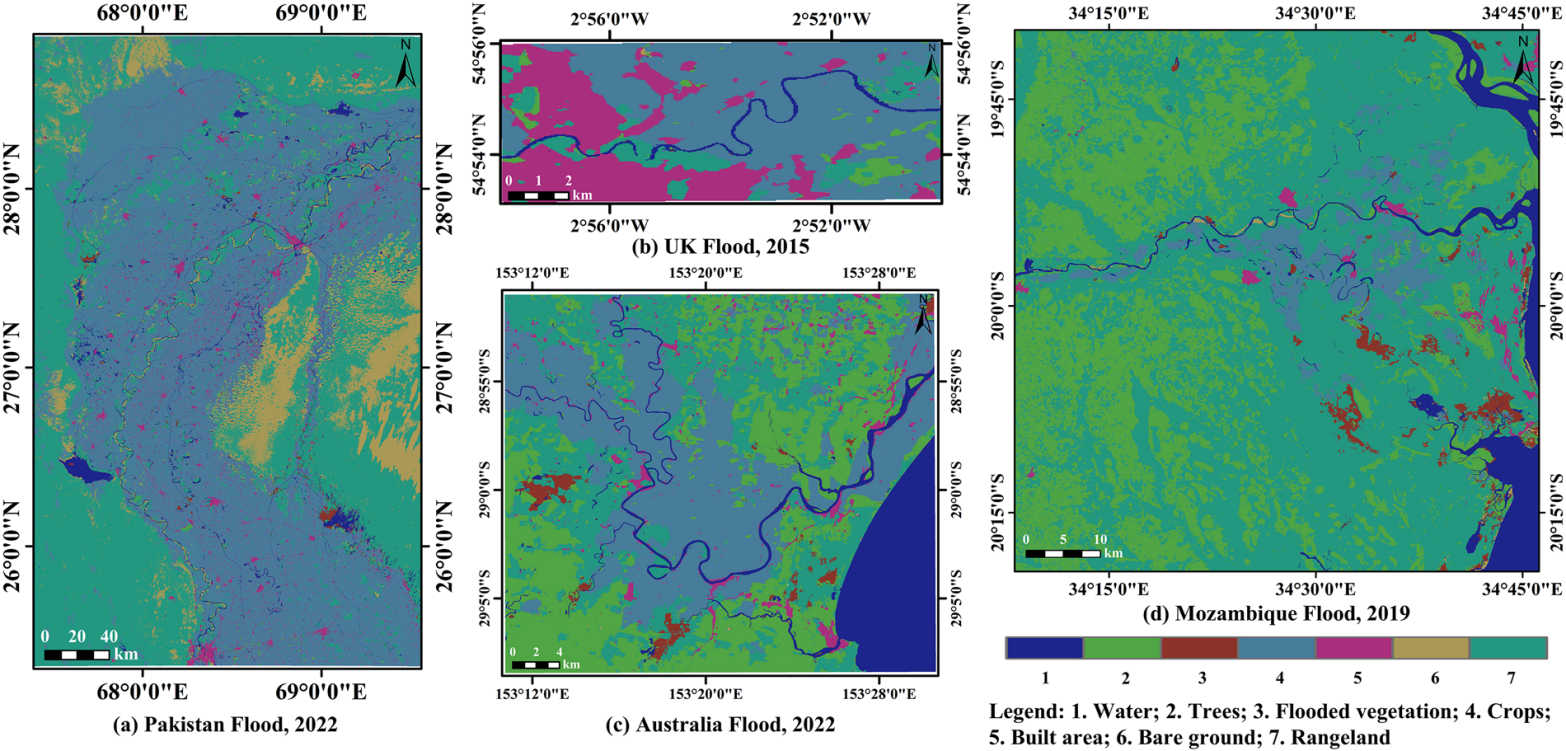
Land use and land cover for (**a**) Pakistan flood 2022, (**b**) UK flood 2015, (**c**) Australia flood 2022, and (**d**) Mozambique flood 2019.

Land cover information is useful for estimating and adjusting friction (Manning coefficient). Land cover information in the study area can be subtracted from the Sentinel-2 land use/land cover dataset^23^. It is produced by a deep learning model by classifying Sentinel-2 data into 9 classes, available at a spatial resolution of up to 10 m for the study area^24^. In the Pakistan study area (Fig. 2(a)), crops are the predominant land cover type, accounting for 46.06% of the total area, while urban areas comprise only 3.01%. In the UK study area (Fig. 2(b)), crops also dominate, constituting 53.47% of the land cover, with urban areas making up 30.41%. In the Australia study area (Fig. 2(c)), trees are the primary land cover type, representing 29.66% of the area, and urban areas account for 2.75%. In the Mozambique study area (Fig. 2(d)), rangeland is the predominant land cover type, covering 56.39% of the area, while urban areas comprise a mere 1.03%.

##### Rainfall data

The rainfall data is sourced from the Integrated Multi-satellite Retrievals for Global Precipitation Measurement (GPM-IMERG) final precipitation products^25^, providing data at a spatial resolution of 0. 1° × 0. 1° and half-hourly temporal resolution^26^. A real-time end-to-end rainfall processing and analysis tool is designed to swiftly obtain rainfall data across diverse spatial resolutions within the designated study area. The tool comprises automatic rainfall crawling, rainfall refinement processing, and rainfall spatio-temporal analysis. Initially, an automatic rainfall crawling process is employed to retrieve and download relevant rainfall data from GPM-IMERG, utilizing a Python-based web crawler. During the refinement stage, bilinear interpolation is employed to resample the data to a temporal resolution of 5 minutes (300 s) and a spatial resolution of 30 m  × 30 m for flood simulations. To ensure alignment with the research area, the data is further refined and extracted using a spatial mask tool. Finally, the temporal and spatial analysis of the rainfall data is conducted to initially determine the time period for flood simulation within the study area, resulting in a grid-based rainfall data at 30 m  × 30 m spatial resolution and 5-minute temporal resolution.

##### Flood measurement data

Flood measurement data includes SAR-based flood maps and surveyed flood outlines. These measurement data are collected for hydrodynamic-based flood inundation calibration and verification. In this study, the SAR-based flood maps for Mozambique, captured at 03:08 Coordinated Universal Time (UTC) on March 20, 2019, and for Australia, at 19:06 UTC on March 2, 2022, are derived from the UrbanSARFloods dataset^27^, with a focus on open flooded areas. These flooded areas is performed by applying an adaptive thresholding algorithm known as the hierarchical split-based change detection approach (HSBA)^28^ to the SAR intensity image pairs. The SAR-based flood map obtained at 01:25 UTC on 30 August 2022 in Pakistan is provided by the Global Flood Awareness System (GloFAS)^29,30^. The spatial resolution of SAR-based flood maps from both the UrbanSARFloods and GloFAS datasets is 20 m. The UrbanSARFloods and GloFAS datasets provide flood mapping outputs in GeoTIFF format, geocoded to the World Geodetic System (WGS) 1984, with latitude and longitude coordinates. In these datasets, flooded areas are indicated by a pixel value of 1, while non-flooded regions are represented by a pixel value of 0. To enhance the reliability and robustness of the UrbanSARFloods dataset, semi-automatic and manual labeling methods are employed. For GloFAS, a widely adopted flood mapping algorithm based on Sentinel-1 data is utilized to ensure data accuracy. Furthermore, the surveyed flood outlines in UK flood 2015 are open to public users under the UK Open Government Licence and can be accessed online^31^. The flood outlines are provided in SHP file format. After downloading SAR-based flood maps and flood outlines, the datasets are cropped to the study areas and reprojected to the Universal Transverse Mercator (UTM) coordinate system for data calibration.

#### Data production

##### Hydrodynamic modeling

In a flood event, water depth is generally much smaller than the horizontal inundation extent, and the flow hydrodynamics can be mathematically described by the 2-D depth-averaged shallow water equations (SWEs)^4,18,32^. SWEs are described by two conservation laws. By neglecting the convective acceleration term, the SWEs for flood modeling can be written as,

1 $$\begin{array}{c}\frac{{\rm{\partial }}h}{{\rm{\partial }}t}+\frac{{\rm{\partial }}{q}_{x}}{{\rm{\partial }}x}+\frac{{\rm{\partial }}{q}_{y}}{{\rm{\partial }}y}=R-I,\frac{{\rm{\partial }}{q}_{x}}{{\rm{\partial }}t}+gh\frac{{\rm{\partial }}(h+z)}{{\rm{\partial }}x}+\frac{g{n}^{2}|q|{q}_{x}}{{h}^{7/3}}=0,\\ \frac{{\rm{\partial }}{q}_{y}}{{\rm{\partial }}t}+gh\frac{{\rm{\partial }}(h+z)}{{\rm{\partial }}y}+\frac{g{n}^{2}|q|{q}_{y}}{{h}^{7/3}}=0,\end{array}$$

where *t* is the time index. *x*, *y* are the spatial horizontal coordinates. *h* is the water height relative to the terrain elevation *z*. *q* = (*q*_*x*_, *q*_*y*_) is the discharge per unit width. *R* represents the rainfall rate, and *I* is the infiltration rate. It is worth noting that the infiltration rate may not be considered when the infiltration has been basically saturated due to continuous rainfall in the study area. *g* is the acceleration due to gravity. *n* is Manning’s friction coefficient. The values of Manning’s friction coefficients are based on the range suggested by FLO-2D User’s Manual^33^ and the land cover types of the study area.

We employ a conventional method, as documented in the references^32,34^, to discretize Eq. (1) from its continuous domain to a discrete domain. This discretization process utilizes a finite difference scheme applied to a staggered grid. It is worth noting that the finite difference scheme is a widely accepted numerical solution technique for simulating flood scenarios and is an integral component of the LISFLOOD-FP^35^, encompassing various forms of SWEs.

The inputs to a hydraulic simulation include an elevation map, grid-based Manning’s friction coefficients, initial conditions, boundary conditions, and the rainfall conditions. As shown in Table 1, for Pakistan flood 2022, the initial conditions mainly consider the water depths obtained from SAR data^4^. Specifically, the SAR-based water depth is determined using an automated Floodwater Depth Estimation Tool (FwDET)^36^, FABDEM, and SAR-based flood mapping from GloFAS^29^ on August 18, 2022. FwDET^36^ enables accurate calculation of floodwater depth using only an inundation map and a DEM. For other flood events, the initial conditions are obtained by prerunning finite difference solver on a dry domain using 3 days of GPM-IMERG rainfall data for UK flood 2015, 2 days of GPM-IMERG rainfall data for Australia flood 2022, and 1 day of GPM-IMERG rainfall data for Mozambique flood 2019. The prerunning duration is primarily dictated by preceding rainfall conditions. Specifically, prolonged rainfall or higher intensity requires an extended prerunning period to ensure accurate initial water levels. Regarding boundary conditions, for Pakistan flood 2022, our main considerations are the inflow boundary of the Indus River and free outflow boundary with a valley slope of 0.2. The inflow boundary from August 18 to August 31, as outlined in Table 2, is derived from hydrological station records along the Indus River in Pakistan, reported by the Government of Pakistan^4^. We meticulously specify the inflow width as spanning 10 pixels (4,800 m for a 480 m resolution), precisely centered on the lowest point of the river. The inflow width may slightly exceed the river width to ensure the stable solution of the inflow boundary. For other flood events, we only utilize free outflow boundary conditions with a valley slope of 0.2. Rainfall is input as a spatial grid, and Manning’s friction coefficients are selected based on land cover (Fig. 2, Table 3). Furthermore, the discrete implementation of finite difference^34^ is systematically applied to the computational grid across the entire study area to simulate the spatiotemporal dynamics of flooding. The discrete implementation^34^ uses two parameters - a weighting factor *θ* that adjusts the amount of artificial diffusion, and a coefficient 0 < *α* ≤1 that is used as a factor by which we multiply the time step. We use the proposed values in^34^, namely *θ* = 0.7, *α* = 0.7. Specifically, when *θ* = 0.7, artificial diffusion most effectively mitigates discontinuities caused by nonlinearities, such as shocks, in the hydrodynamic system. Simultaneously, when *α* = 0.7, the time step can be optimally regulated, thereby substantially improving the stability of the numerical model.

**Table 1:** Input details to hydrodynamic simulations of different flood events.

Flood Event	Area and Duration	Initial Condition	Boundary Condition	DEM	Rainfall	Manning
Pakistan Flood 2022	85, 616.5 km^2^, 14 days (from August 18 to August 31, 2022)	SAR-based water depth	Inflow boundary (Table 2) + Free (valley slope)	FABDEM	GPM-IMERG	Table 3
UK Flood 2015	135.5 km^2^, 3 days (from December 4 to December 7, 2015)	Prerunning on a dry domain using 3-day rainfall data	Free (valley slope)	FABDEM	GPM-IMERG	Table 3
Australia Flood 2022	1, 361.3 km^2^, 10 days (from February 20 to March 02, 2022)	Prerunning on a dry domain using 2-day rainfall data	Free (valley slope)	FABDEM	GPM-IMERG	Table 3
Mozambique Flood 2019	6, 190.9 km^2^, 6 days (from March 14 to March 20, 2019)	Prerunning on a dry domain using 1-day rainfall data	Free (valley slope)	FABDEM	GPM-IMERG	Table 3

**Table 2:** Daily discharges for inflow boundary of Pakistan flood 2022.

Dates	18-Aug	19-Aug	20-Aug	21-Aug	22-Aug	23-Aug	24-Aug
Discharge at inflow (*m*^3^/*s*)	9345	9798	10251	11667	13677	15914	15489
Dates	25-Aug	26-Aug	27-Aug	28-Aug	29-Aug	30-Aug	31-Aug
Discharge at inflow (*m*^3^/*s*)	14272	13875	13875	13734	14187	14527	14696

**Table 3:** Manning’s coefficient values based on land cover types.

Land cover	Water	Trees	Flooded vegetation	Crops	Built area	Bare ground	Rangeland
Manning’s coefficients	0.0350	0.1200	0.0800	0.0350	0.3750	0.0265	0.0375

We implement the numerical solution using Python. We obtain results at 30 m  × 30 m spatial resolution. Temporal resolution adheres to the Courant-Friedrichs-Lewy (CFL) condition for hyperbolic system stability. Due to the fine temporal resolution (less than 10 seconds), the 14-day simulations for Pakistan flood 2022 are executed on an NVIDIA A6000 GPU, completing in approximately two weeks. The 3-day simulations for UK flood 2015 require about 4 hours. The 10-day simulations for Australia flood 2022 take approximately two days to complete, while the 6-day simulations for Mozambique flood 2019 on the same GPU also require about two days.

##### FloodCastBench Datasets

**Fig. 3:**
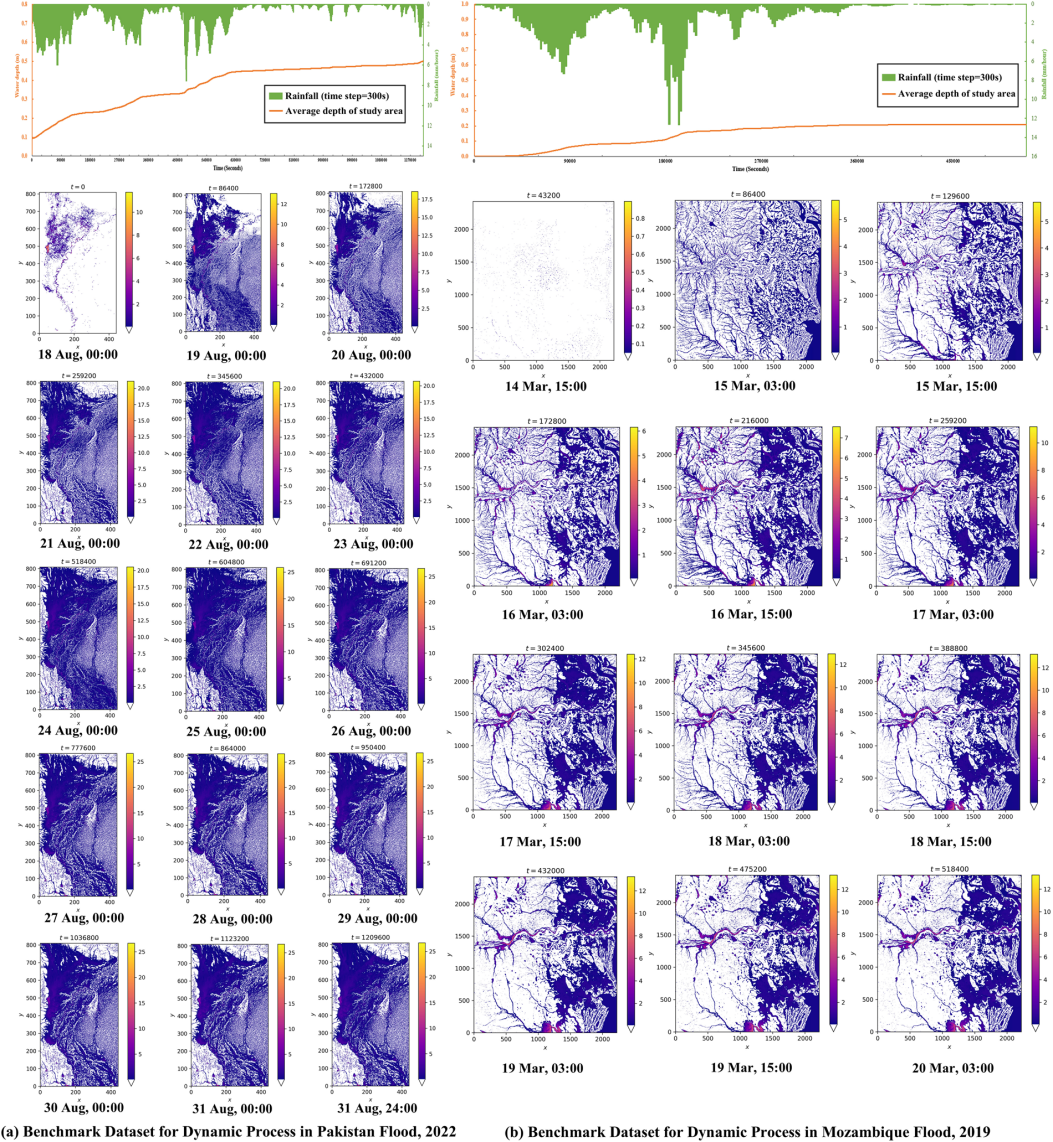
Benchmark Datasets for dynamic process in Pakistan flood 2022 and Mozambique flood 2019.

**Fig. 4:**
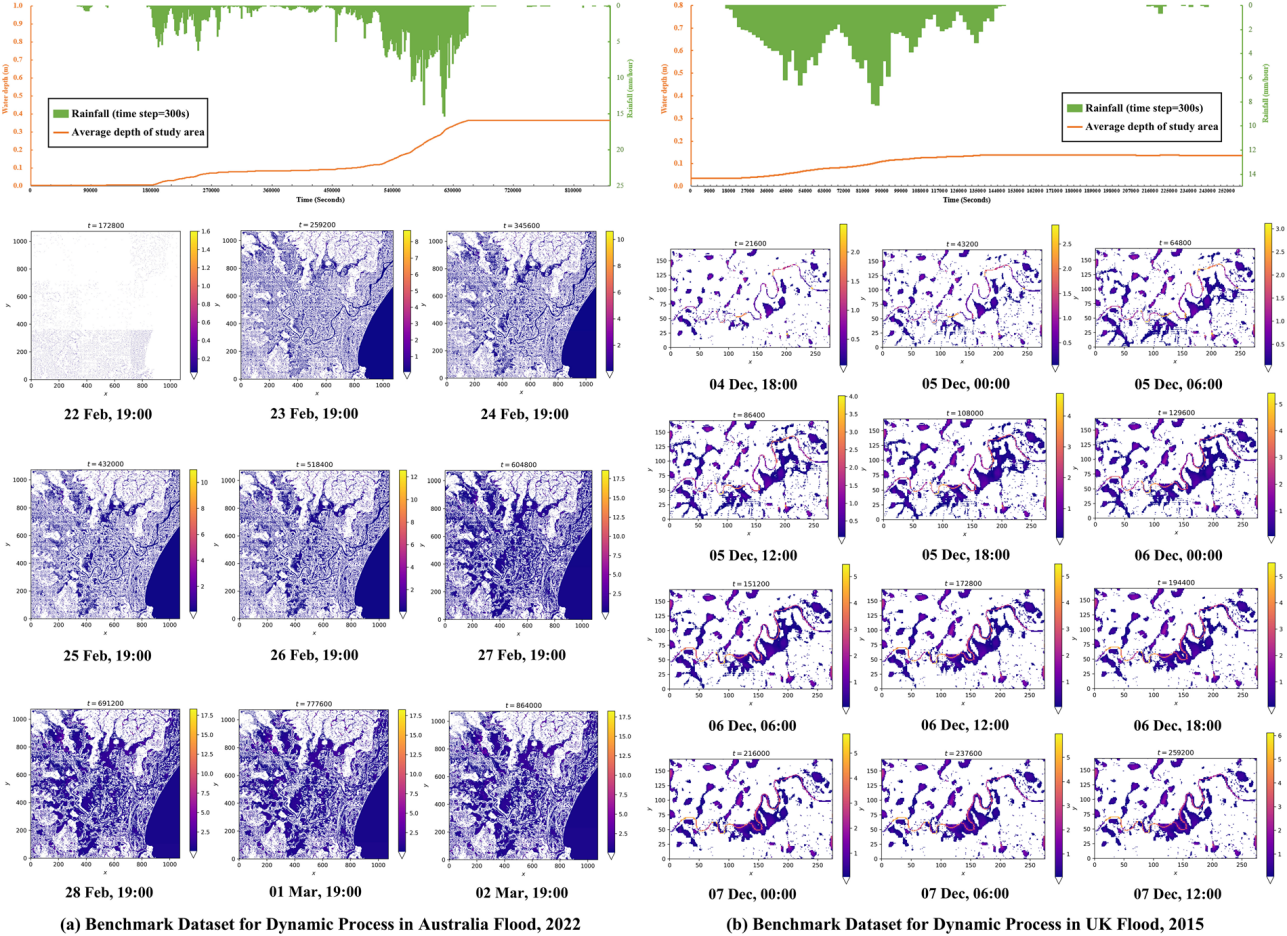
Benchmark Datasets for dynamic process in Australia flood 2022 and UK flood 2015.

Finally, our benchmark dataset for flood forecasting (FloodCastBench) can be generated, as illustrated in Figs. 3 and 4. All flood dynamics datasets have a spatial resolution of 30 m  × 30 m and a temporal resolution of 300 seconds. This benchmark dataset comprises two categories: extremely large-scale floods (Fig. 3), including Pakistan flood 2022 and Mozambique flood 2019, are used to develop low-fidelity flood forecasting ML models; and large-scale floods (Fig. 4), including Australia flood 2022 and UK flood 2015, are used to develop high-fidelity flood forecasting ML models. The curves above Figs. 3 and 4 illustrate changes in the average water depth in the study area in response to rainfall. It is evident that the continuous rainfall leads to a sustained increase in regional water depths. These observed trends serve as preliminary confirmation of the reliability of our benchmark dataset. Below Figs. 3 and 4, the spatial distributions of flood depths over time in the study area are illustrated. Furthermore, the FloodCastBench datasets are verified and calibrated by comparing the final flood inundations with flood measurement data.

#### Data calibration

To achieve reliable flood dynamics simulations, we calibrate the hydrodynamic model parameters using flood measurement data. After multiple trials with different terrains (e.g., FABDEM^22^ or Shuttle Radar Topography Mission (SRTM) 30 m^37^) and varying Manning coefficients, the final parameters of the calibrated hydrodynamic model are presented in Table 1. A comparative analysis of calibrated flood inundation extents from the FloodCastbench dataset and flood measurement data is presented in the Technical Validation section.

## Data records

The FloodCastBench dataset has been uploaded in Zenodo^38^. It is designed to be open and accessible to all researchers in the fields of flood, hydrology, and ML. This dataset comprises three folders: the low-fidelity flood forecasting folder, the high-fidelity flood forecasting folder, and the relevant data folder. The low-fidelity flood forecasting folder includes data on the 2022 Pakistan flood and the 2019 Mozambique flood, both with a spatial resolution of 480 m. The high-fidelity flood forecasting folder contains two subfolders: one for the 2022 Australia flood and the 2015 UK flood with a spatial resolution of 30 m, and another for the same floods with a spatial resolution of 60 m. Notably, for the Pakistan flood, given the extensive study area, a computationally efficient approach is employed to achieve the target resolution. Specifically, input parameters such as terrain and rainfall are first downsampled to a resolution of 480 m using bilinear interpolation and then directly processed by the hydrodynamic model. For other flood events, to ensure high accuracy, all results are initially generated at a 30 m resolution and subsequently resampled to target resolutions, such as 480 m and 60 m, using bilinear interpolation. All data files are stored in TIFF format, with a temporal resolution of 300 seconds, and file names are numbered sequentially, incremented every 300 seconds until the simulation endpoint. The relevant data folder includes five subfiles: DEM, land use and land cover, rainfall data, georeferenced files for the four flood events, and initial condition files for both low- and high-fidelity flood simulations. The DEM, land use and land cover, rainfall, and initial condition data are all provided in TIFF format. The rainfall data is organized in a format of year-month-day-hour-minute-second. Georeferenced files provide geographic extent and spatial reference to support viewing and analysis of the associated TIFF files in GIS. To provide an overview of the dataset, Figs. 3 and 4 visualize the spatiotemporal process of floods in the study areas.

## Technical Validation

### Validation of the FloodCastBench dataset by flood measurement data

**Fig. 5:**
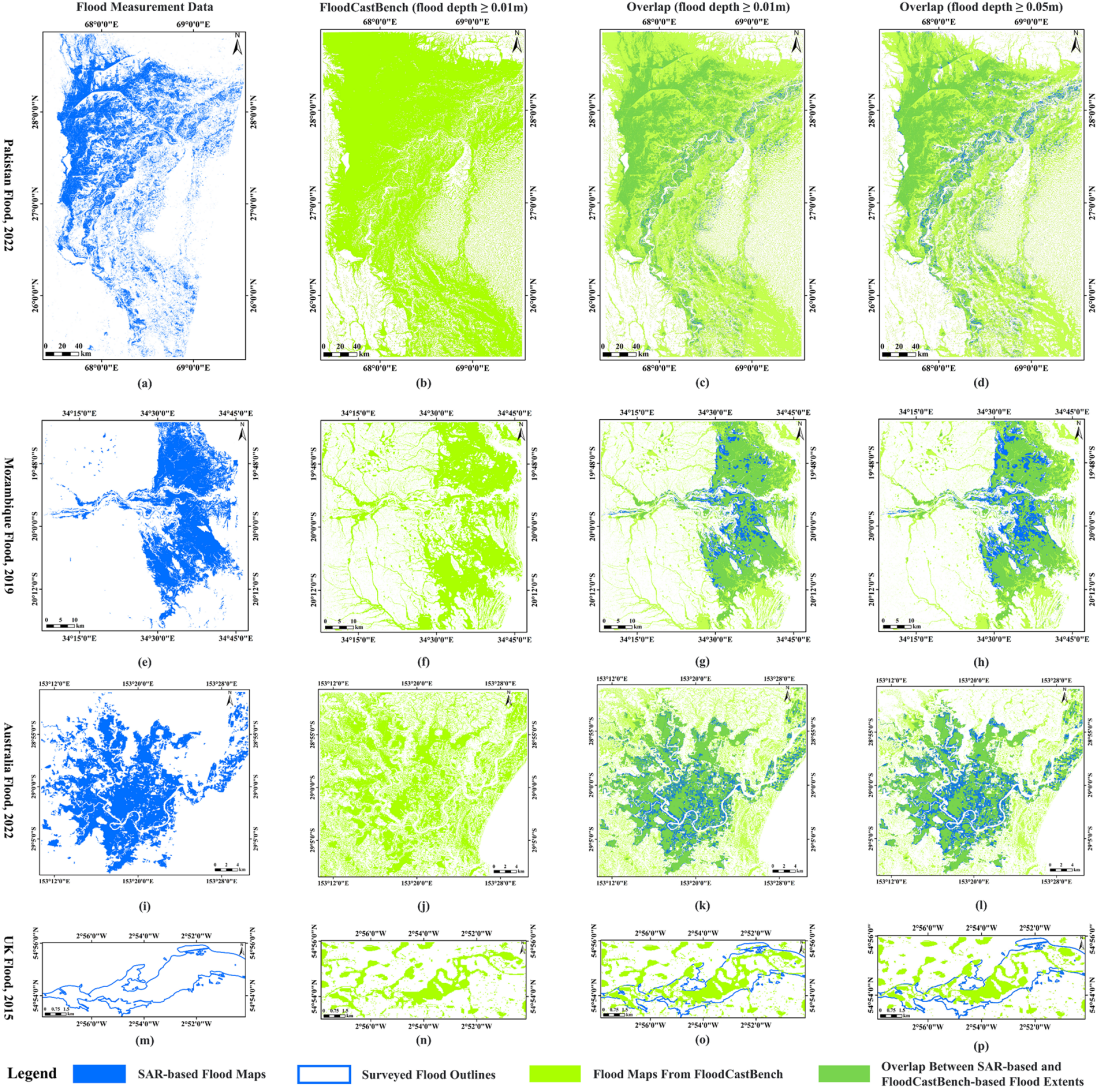
Flood extent results and comparative analysis. (**a**) SAR-based flood map on 30 August 2022 in Pakistan. (**b**) FloodCastBench-based flood map at 01:25 on 30 August 2022 in Pakistan (flood depth ≥ 0.01m). (**c**) Comparison between SAR-based and FloodCastBench-based flood maps (flood depth ≥ 0.01m) in Pakistan, where the CSI is 0.295710. (**d**) Comparison between SAR-based and FloodCastBench-based flood maps (flood depth ≥ 0.05m) in Pakistan, where the CSI is 0.327213. (**e**) SAR-based flood map on 20 March 2019 in Mozambique. (**f**) FloodCastBench-based flood map at 03:00 on 20 March 2019 in Mozambique (flood depth ≥ 0.01m). (**g**) Comparison between SAR-based and FloodCastBench-based flood maps (flood depth ≥ 0.01m) in Mozambique, where the CSI is 0.546789. (**h**) Comparison between SAR-based and FloodCastBench-based flood maps (flood depth ≥ 0.05m) in Mozambique, where the CSI is 0.548720. (**i**) SAR-based flood map on 2 March 2022 in Australia. (**j**) FloodCastBench-based flood map at 19:00 on 2 March 2022 in Australia (flood depth ≥ 0.01m). (**k**) Comparison between SAR-based and FloodCastBench-based flood maps (flood depth ≥ 0.01m) in Australia, where the CSI is 0.596893. (**l**) Comparison between SAR-based and FloodCastBench-based flood maps (flood depth ≥ 0.05m) in Australia, where the CSI is 0.593301. (**m**) Surveyed flood outlines in UK. (**n**) FloodCastBench-based flood map at 12:00 on 7 December 2015 in UK (flood depth ≥ 0.01m). (**o**) Comparison between surveyed flood outlines and FloodCastBench-based flood maps (flood depth ≥ 0.01m) in UK, where the CSI is 0.358639. (**p**) Comparison between surveyed flood outlines and FloodCastBench-based flood maps (flood depth ≥ 0.05m) in UK, where the CSI is 0.313800.

Figure 5 shows the flood inundation extents from FloodCastBench at a specific time and a comparative analysis with flood measurement data. The timing of the FloodCastBench-based flood maps is closely aligned with that of the flood measurement data. The FloodCastBench inundation extents (second column in Fig. 5) are defined for flood depths exceeding 0.01 m. Notably, the FloodCastBench inundation extents in Pakistan, Mozambique, and Australia cover most of the SAR-based flood maps. Additionally, the riverside flood areas identified by FloodCastBench in UK match the surveyed flood outlines. To further validate results from FloodCastBench, we performed overlapping assessments at various flood depth thresholds (third and fourth columns in Fig. 5). Furthermore, the critical success index (CSI) is considered as a quantitative measure of the spatial discrepancies between SAR-based and FloodCastBench-based flood maps across different thresholds. The calculation of CSI is presented in Eq. (5). The FloodCastBench-based inundation extents encompass a significant portion of the SAR-based area and surveyed flood outlines, aligning their overall flood extents. However, a comparative assessment in Mozambique (Fig. 5(g)) and Australia (Fig. 5(k)) reveals discrepancies between the SAR-based and FloodCastBench-based flood extents, particularly in crop areas. This may be due to the hydrodynamic model not accounting for different crop types and uncertainty of rainfall in GPM-IMERG data^39^. Furthermore, by comparing FloodCastBench-based flood extents across various flood depth thresholds with SAR-based flood extents for the Pakistan flood, as shown in Fig. 5 (c) and (d), we find that areas of non-congruence with SAR-based extents are predominantly crop areas. This discrepancy likely results from flood depths not exceeding crop heights, leading to minimal changes in backscattering and the subsequent absence of flood detection in SAR imagery^4,40^. Consequently, SAR-based flood extents align more closely at higher thresholds for the Pakistan flood, as indicated by a higher CSI when the flood depth ≥ 0.05m compared to the flood depth ≥ 0.01m. For other flood events, FloodCastBench-based extents consistently correlate well with flood measurement data across different flood depth thresholds. These comparative results further validate the effectiveness of the FloodCastBench dataset.

### Validation of the FloodCastBench dataset for machine learning

To validate the effectiveness of the FloodCastBench dataset for ML, we develop a flood forecasting task and benchmark to assess the performance and transferability of ML-based foundation models at different scales. We categorize the flood forecasting task into low-fidelity and high-fidelity forecasts. Low-fidelity flood forecasting utilizes datasets from the Pakistan and Mozambique floods, with a spatial resolution of 480m and a temporal resolution of 300 seconds. High-fidelity flood forecasting uses datasets from the Australia and UK floods, with spatial resolutions of 60m or 30m and the same temporal resolution of 300 seconds. In both tasks, we forecast flood depth from time *t*=1 to *t*=20.

For low-fidelity forecasting, we validate the model on the Pakistan flood 2022 and tested its transferability on the Mozambique flood 2019. The Pakistan flood dataset spans 14 days, with data recorded at 300-second intervals. Each sample consists of 20 time steps, resulting in a total of 201 samples. These samples are partitioned into training (first 10 days, 145 samples), validation (11th and 12th days, 28 samples), and testing (final 2 days, 28 samples) sets. For cross-regional forecasting, the Mozambique flood dataset covers a period of 6 days, yielding 86 samples, with each sample containing 20 time steps recorded at 300-second intervals. For high-fidelity forecasting, we validate the model on the Australia flood 2022 and test its transferability on the UK flood 2015. The Australia dataset spans 10 days, recorded at 300-second intervals, with each sample comprising 20 time steps, resulting in a total of 144 samples. These samples are divided into training (first 8 days, 116 samples), validation (9th day, 14 samples), and testing (10th day, 14 samples) sets. The UK flood dataset, which spans 3 days, is utilized for cross-regional forecasting, providing 43 samples, with each sample also containing 20 time steps recorded at 300-second intervals.

**Fig. 6:**
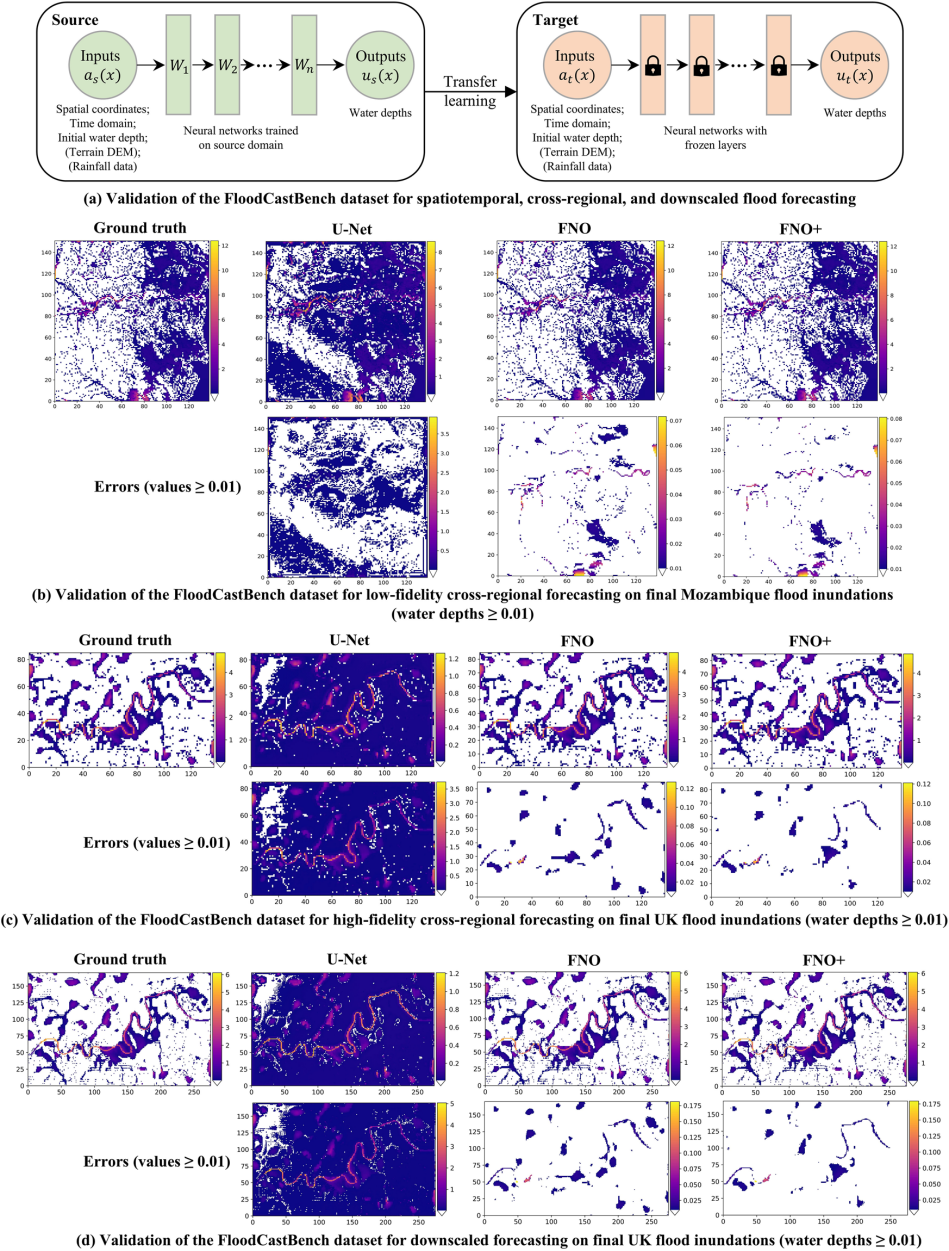
Validation of the FloodCastBench dataset for machine learning.

Furthermore, we establish a flood forecasting benchmark using commonly employed neural networks. Specifically, we use the following models: **U-Net:** An architecture widely used for image-to-image regression tasks, employing four blocks of 3D convolutions and deconvolutions^41^. **Fourier Neural Operator (FNO):** Utilizing an oneshot training strategy with direct space-time convolutions^4,42,43^. As illustrated in Fig. 6(a), for both U-Net and FNO, the inputs *a*(*x*) are spatial coordinates (*X*, *Y*), the time domain (*T*), and initial water depth at *t*=1. **FNO+:** Similar to FNO, the primary distinction is in the input. In addition to the inputs utilized in FNO, FNO+ incorporates additional physical variables, including terrain DEM and time series rainfall data from *t*=1 to *t*=20, as indicated in the brackets in Fig. 6(a). The output *u*(*x*) for all models corresponds to the water depth for the subsequent 19 time steps, ranging from *t*=2 to *t*=20. We employ 4 Fourier layers for FNO, truncating the transform to the 12 lowest Fourier modes, with a latent space dimension of 20. For U-Net, the feature dimension of the first layer is set to 32. All models are trained with a batch size of 1 for 100 epochs, using a cosine learning rate scheduler starting at 0.001 and decaying to 0. The Adam optimizer, with *β*_1_ = 0.9, *β*_2_ = 0.999, and a weight decay of 10^−4^ is used. The models are implemented in PyTorch and trained on a single NVIDIA RTX A40 48GB GPU.

Flood forecasting is a spatiotemporal prediction task. To comprehensively evaluate the performance of the benchmark models, we use relative mean square error (RMSE), Nash-Sutcliffe efficiency coefficient (NSE), and Pearson correlation coefficient (*r*):

2 $${\rm{RMSE}}=\frac{1}{N}\mathop{\sum }\limits_{i=1}^{N}\frac{{| {y}_{i}-{p}_{i}| }^{2}}{{| {y}_{i}| }^{2}},$$

3 $${\rm{NSE}}=1-\frac{{\sum }_{i}^{N}{| {y}_{i}-{p}_{i}| }^{2}}{{\sum }_{i}^{N}{| {y}_{i}-{\bar{y}}_{i}| }^{2}},$$

4 $$r=\frac{{\sum }_{i}^{N}({y}_{i}-{\bar{y}}_{i})({p}_{i}-{\bar{p}}_{i})}{\sqrt{{\sum }_{i}^{N}{({y}_{i}-{\bar{y}}_{i})}^{2}{\sum }_{i}^{N}{({p}_{i}-{\bar{p}}_{i})}^{2}}},$$

where *y*_*i*_ is the true flood depths and *p*_*i*_ is the predicted flood depths. We also consider the CSI that measures the spatial accuracy of the classification of cells as flooded on non-flooded areas for a given flooding threshold *γ*. CSI is evaluated as follows:

5 $${\rm{CSI}}=\frac{{\rm{TP}}}{{\rm{TP}}+{\rm{FP}}+{\rm{FN}}},$$

where TP represents true positives (cells where both predictions and ground truths exceed *γ*), FP represents false positives (cells where ground truths are below *γ* but predictions exceed *γ*), and FN represents false negatives (cells where the model fails to predict a flooded area). In our experiments, we set *γ* to {0.001 m, 0.01 m} to account for very shallow waters.

#### Validation of the FloodCastBench dataset for spatiotemporal flood forecasting

Table 4 presents the low-fidelity and high-fidelity flood forecasting results for predicting over 20 time steps, using true water depth values at time *t* = 0 as the initial input. The FNO model captures the dynamics of water flows more accurately than U-Net. Specifically, in low-fidelity flood forecasting, FNO reduces the test error (RMSE) by 97.19% compared to U-Net, and in high-fidelity flood forecasting, the reduction is 99.25%. Additionally, FNO demonstrates better spatial accuracy (higher CSI) under both the low threshold (*γ* = 0.001) and the high threshold (*γ* = 0.01). Importantly, FNO+ outperforms all models in low-fidelity and high-fidelity flood forecasting, improving performance compared to FNO. For instance, in low-fidelity flood forecasting, FNO+ reduces the test error, as measured by RMSE, by 5.60% compard to FNO, while in high-fidelity flood forecasting, the reduction increases to 7.44%. Furthermore, FNO+ exhibits superior spatial accuracy, attaining the highest CSI across various thresholds. This highlights that incorporating physical factors (such as rainfall and terrain) can enhance model performance. Although Table 4 only includes commonly used methods, it provides a solid benchmark and detailed evaluation criteria for foundational models in flood spatiotemporal prediction tasks. Furthermore, it validates the effectiveness of the FloodCastBench dataset in supporting ML models for flood forecasting.

**Table 4:** Results of foundation models for low-fidelity and high-fidelity flood forecasting.

Tasks	Methods	RMSE↓	NSE↑	*r*↑	CSI↑	
					*γ* = 0.001	*γ* = 0.01
Low-fidelity (480m)	U-Net	0.079399	0.991705	0.995934	0.815218	0.809986
	FNO	0.002232	0.999993	**0.999997**	0.966631	0.992669
	FNO+	**0.002107**	**0.999994**	**0.999997**	**0.976716**	**0.993993**
High-fidelity (60m)	U-Net	0.566626	0.560984	0.942675	0.779631	0.837399
	FNO	0.004258	0.999975	0.999987	0.895553	0.980748
	FNO+	**0.003941**	**0.999979**	**0.999990**	**0.939638**	**0.984588**

#### Validation of the FloodCastBench dataset for cross-regional and downscaled flood forecasting

Table 5 presents the transferability of models trained on the Pakistan flood directly to low-fidelity Mozambique flood forecasting, and models trained on the Australia flood directly to high-fidelity UK flood forecasting and downscaled to UK flood prediction with a spatial resolution of 30 m. The model’s transferability is achieved through transfer learning, enabling the direct application of a model trained on a source dataset to a target dataset. As shown in Fig. 6(a), when transferred to a different target domain (such as a different region or grid resolution), the weights of the model, pre-trained on the source domain, remain frozen. The only adjustment required is modifying the input corresponding to the target domain. For low-fidelity flood forecasting (a spatial resolution of 480 m), U-Net achieves poor transfer results due to the significant domain shift between Pakistan and Mozambique. However, FNO and FNO+ achieve excellent model transfer results. In high-fidelity flood forecasting (a spatial resolution of 60 m), FNO and FNO+ also outperform U-Net, achieving impressive transferred results. Additionally, comparing FNO and FNO+ in both low-fidelity and high-fidelity flood forecasting, FNO+ demonstrates superior accuracy. For example, in low-fidelity flood forecasting, FNO+ reduces the RMSE by 51.85% and improves the NSE by 52.57% compared to FNO. In high-fidelity flood forecasting, FNO+ achieves a 14.55% lower RMSE and a 0.04% higher NSE than FNO. This demonstrates that incorporating physical variables enhances model transferability. Furthermore, for zero-shot downscaled flood forecasting (a spatial resolution of 30 m), both FNO and FNO+ exhibit excellent performance. Additionally, the final flood inundation results for various methods are visualized and compared across three scenarios: low-fidelity cross-regional flood forecasting in Mozambique, high-fidelity cross-regional flood forecasting in the UK, and zero-shot downscaled flood forecasting in the UK, as shown in Fig. 6(b),(c), and (d), respectively. It is evident that the FNO and FNO+ models exhibit superior spatial prediction performance compared to U-Net. These experiments and results further validate the effectiveness of the FloodCastBench dataset in evaluating cross-regional and downscaled flood forecasting models. Notably, while transfer learning demonstrates effectiveness with the FloodCastBench dataset, this experiment primarily aims to evaluate performance at a time step of *t*=20. For predicting floods with longer time steps, especially when the target domain significantly differs from the source domain, a small amount of training data from the target domain is required to fine-tune the model to ensure rapid adaptation.

**Table 5:** Model transferred results in low-fidelity and high-fidelity flood forecasting, as well as zero-shot downscaling.

Tasks	Methods	RMSE↓	NSE↑	*r*↑	CSI↑	
					*γ* = 0.001	*γ* = 0.01
Low-fidelity (480m)	U-Net	1.602850	-72.350318	0.903450	0.724442	0.667776
	FNO	0.163300	0.453167	0.980383	0.892473	**0.915509**
	FNO+	**0.078633**	**0.955450**	**0.985521**	**0.934028**	0.912712
High-fidelity (60m)	U-Net	0.525467	0.690026	0.956584	0.314035	0.517440
	FNO	0.028990	0.998575	0.999600	0.755766	0.925626
	FNO+	**0.024771**	**0.998949**	**0.999645**	**0.859821**	**0.963278**
High-fidelity (30m)	U-Net	0.556424	0.660375	0.960544	0.282739	0.553059
	FNO	0.028297	0.998706	0.999604	0.722694	0.927164
	FNO+	**0.025190**	**0.998953**	**0.999639**	**0.810674**	**0.958525**

## Usage Notes

In this work, we introduce the FloodCastBench dataset, a large-scale resource designed for flood modeling and forecasting using ML techniques. This dataset aims to provide comprehensive low-fidelity and high-fidelity flood forecasting datasets. However, it is important to recognize its limitations. Specifically, the uncertainty of the flood dynamics data needs further validation through alternative time-series hydrological data. Additionally, the flood forecasting task is currently limited to 20 time steps due to GPU memory constraints. For extended forecasts, such as 14 consecutive days in Pakistan flood 2022 or 10 consecutive days in Australia flood 2022, using GPUs with larger memory capacities or employing sequence-to-sequence neural forecasting methods^4^ is recommended.

Our vision for FloodCastBench is to establish it as a foundational, dynamically expanding community flood forecasting dataset. We aim for it to be accessible and augmentable by researchers in the hydrology and ML fields. At present, the spatial distribution of flood events in FloodCastBench is restricted to a few regions globally. We hope that users will contribute and share their data, allowing FloodCastBench to eventually cover a wide range of flood events worldwide, as depicted in Fig. 1(a).

## Data Availability

The code that was used to produce the FloodCastBench dataset is available at https://github.com/HydroPML/FloodCastBench.
